# Older Adults' Perceptions of College Campus Accessibility

**DOI:** 10.1093/geroni/igab046.2814

**Published:** 2021-12-17

**Authors:** Ashley Ermer, Nadine Verna

**Affiliations:** Montclair State University, Montclair, New Jersey, United States

## Abstract

The emerging Age-Friendly University Global Network encourages universities to engage older adults in university activities (Gerontological Society of America, 2019). As such, attention should be devoted to the accessibility of campus facilities to older adults as a potential mechanism to increase age diversity. Intergenerational interactions, which may take place on college campuses, promote better perceptions of other generations (Bertram et al., 2017), making campus accessibility for all age groups a priority. The present study sought to uncover older adults’ perceptions of campus accessibility via an online survey. Participants were recruited through local newsletters, word of mouth, and included 81 community members (Age mean=71.58 years; 79% female; 89% White; 43% traveled to campus every few months). Descriptive analyses were conducted for closed-ended responses and two members of the research team used a constant comparative method (Corbin & Strauss, 2015) to code open-ended responses. Participants felt that campus was somewhat accessible (M = 2.72;1(very inaccessible) to 5(very accessible)), moderately easy to walk around (M=3.79;1(extremely difficult) to 7(extremely easy)), and felt somewhat welcome on campus (M=3.27; 1(strongly disagree) to 7(strongly agree). The following general themes emerged in the open-ended responses: 1)inaccessibility on campus was due to parking, drop-off locations, and topography (e.g., due to stairs, distance, hills) constraints; 2)feeling welcome on campus was due to people being helpful; and 3)difficulty in attending events was due to parking and lack of knowledge about events. Implications for campus initiatives that aim to attract older adults, especially for campuses that have topography constraints, will be discussed.

